# Emotional Intelligence and Job Satisfaction Among Staff Nurses: A Descriptive Survey

**DOI:** 10.7759/cureus.81810

**Published:** 2025-04-06

**Authors:** Nilima R Bhore, Archana R Dhanawade

**Affiliations:** 1 Obstetrics and Gynecological Nursing, Bharati Vidyapeeth (Deemed to be University) College of Nursing Sangli, Sangli, IND

**Keywords:** assess, emotional intelligence, hospital environment, hospital staff, job satisfaction, staff nurses

## Abstract

Introduction: Emotional intelligence and job satisfaction have become increasingly important in today’s workplace, offering significant benefits in personal and professional development. However, there is limited research available on these topics within the Indian healthcare context, especially regarding nurses. Specifically, this paper seeks to assess nurses' emotional intelligence levels, evaluate their job satisfaction, and determine how emotional intelligence influences workplace satisfaction. Emotional intelligence is essential for nurses in managing stress, fostering effective patient interactions, and maintaining a positive work environment. Likewise, job satisfaction impacts motivation, retention, and overall performance. By exploring these factors, the study aims to provide insights into their interconnectedness and contribute to strategies that enhance nurse well-being and improve healthcare service quality.

Methodology: A descriptive research design with a quantitative approach was employed in this study. Using a simple random sampling technique, 100 staff nurses were selected as participants. To assess emotional intelligence and job satisfaction, the study utilized the Emotional Intelligence Inventory Scale and the Minnesota Job Satisfaction Questionnaire. The researcher administered the questionnaire to the staff nurses, allowing them 15-20 minutes to complete the questions. Data analysis was performed using descriptive and inferential statistics to summarize the findings and identify relationships between emotional intelligence and job satisfaction. This approach provided a comprehensive understanding of the nurses' emotional intelligence and job satisfaction levels.

Results: Among 100 staff nurses, the majority (58%; n=58) were aged 20-30 years, were female (70%; n=70), were married (64%; n=64%), and had 1-10 years of hospital experience (68%; n=68). Most (60%; n=60) earned a monthly salary of Rs. 10,000-20,000, and (52%; n=52) had GNM qualifications. A majority of 79 (79%) worked as staff nurses, with 47 (47%) in general wards. Emotional intelligence levels were very high among 77 (77%) nurses, while 63 (63%) were highly satisfied with their jobs. The mean emotional intelligence score was 191.59 (S.D. 18.0264), and the mean job satisfaction score was 75.14 (S.D. 11.9974). A significant positive correlation (r = 0.4059, p = 0.000002) was observed between emotional intelligence and job satisfaction.

Conclusion: The findings suggest that nurses with higher emotional intelligence tend to experience greater job satisfaction. This highlights the critical role emotional intelligence plays in influencing job satisfaction levels. By promoting emotional intelligence in nursing practice, hospital management can create a more positive work environment, which may lead to improved nurse satisfaction, better patient care, and reduced turnover rates. The results emphasize the importance of investing in emotional intelligence development as a key strategy for enhancing nurse well-being and overall hospital performance.

## Introduction

Emotional intelligence enables nurses to manage stress, build strong patient relationships, and maintain a positive work environment, directly influencing job satisfaction. While studies highlight this connection, research on Indian nurses remains limited. This study explores how emotional intelligence affects job satisfaction, addressing gaps, and improving healthcare workplace strategies.

A study by Ibrahim et al. explored the relationship between emotional intelligence and clinical performance among nursing students during obstetrics and gynecologic practical training. The findings revealed a significant positive correlation between emotional intelligence and students’ clinical performance. Students with higher emotional intelligence demonstrated better communication, critical thinking, and practical skills, contributing to improved patient care and clinical outcomes. This study emphasizes the importance of integrating emotional intelligence development into nursing education programs. Enhancing emotional intelligence through targeted training and support can foster professional growth, improve performance, and equip nursing students to handle clinical challenges effectively [1].

A study by Mazumder et al. examined the relationships between organizational commitment, supervisory support, and job satisfaction among nurses in a public specialized hospital in Bangladesh. The findings revealed a significant positive correlation between organizational commitment, supervisory support, and job satisfaction. Nurses who received strong supervisory support and demonstrated higher organizational commitment reported greater job satisfaction. The study highlights the crucial role of supportive supervision and organizational policies in fostering a positive work environment. Encouraging commitment through effective leadership and support can enhance job satisfaction, leading to improved performance and retention among nursing staff in healthcare settings [2].

A study by Abu Safieh et al. investigated the interplay between job-related stress, quality of work life, and quality of nursing care among critical care nurses. The findings revealed that elevated job-related stress negatively impacted both the quality of work life and nursing care provided by the participants. Conversely, a higher quality of work life was associated with better nursing care outcomes. The study emphasizes the importance of addressing stress management and improving workplace conditions for critical care nurses. Implementing strategies to enhance work-life quality can significantly reduce stress levels and improve the overall quality of nursing care delivered [3].

A study by Munyug et al. explored the relationship between emotional intelligence and job satisfaction among state civil servants. The findings demonstrated a significant positive correlation, indicating that individuals with higher emotional intelligence were more likely to experience job satisfaction. Emotional intelligence was found to enhance interpersonal relationships, stress management, and decision-making, all of which contribute to a more fulfilling work experience. The study highlights the importance of fostering emotional intelligence through targeted training programs to improve job satisfaction and employee performance. This approach can benefit organizations by promoting a motivated, efficient, and satisfied workforce [4].

Researching emotional intelligence and job satisfaction among nurses is crucial as emotional intelligence enhances patient care, reduces stress, fosters teamwork, and improves job performance. Higher emotional intelligence leads to better communication, lower burnout, and increased retention, ultimately strengthening healthcare efficiency and ensuring a more supportive and effective work environment for nurses. It helps identify factors influencing their job contentment, which can impact patient care and overall healthcare quality. Additionally, understanding emotional intelligence contributes to better team dynamics, communication, and coping mechanisms in a demanding healthcare environment, ultimately enhancing the well-being of both nurses and patients.

## Materials and methods

A total of 100 staff nurses working in various healthcare settings were selected as the study sample. Simple random sampling was used to ensure that every nurse had an equal chance of being selected, reducing selection bias and increasing the reliability of the results. This method involves randomly choosing participants from a larger population, ensuring that the sample accurately represents the broader group. To implement simple random sampling, a list of eligible nurses was created, and participants were selected using a random process, such as a lottery system or a computer-generated random number table. This approach prevents favouritism or unintentional bias, making the study more objective. By using simple random sampling, the study findings can be generalized more effectively to similar populations. This method enhances the study’s validity by ensuring diversity in experiences, work environments, and individual characteristics, providing a more accurate understanding of emotional intelligence and job satisfaction among nurses.

The study was approved by the Institutional Ethics Committee of Bharati Vidyapeeth (Deemed to be University), College of Nursing, Sangli (Reg. No. EC/NEW/INST/2024/MH/0414). All participants were fully informed about the study's purpose, procedures, and their right to withdraw at any time without consequences, and written consent was obtained. The study included nurses who were currently working in hospitals and had at least one year of experience. Data was collected using a structured questionnaire. The tool had two sections: the first focused on demographic details such as age, gender, qualifications, and experience, while the second included questions related to the study's objectives. The questionnaire was designed to ensure clarity and collect accurate, relevant information effectively.

The researcher administered the questionnaires to the 100 staff nurses. The sample selection criteria for the study included nurses working in hospitals with a minimum of one year of work experience, ensuring that they had sufficient exposure to the demands of the profession. Nurses who did not provide written consent for participation were excluded from the study. This approach ensured that the participants had relevant experience while respecting their autonomy and willingness to take part in the research. Each participant was provided with 15-20 minutes to respond to the items on the Emotional Intelligence Inventory Scale and the Minnesota Job Satisfaction Questionnaire. The Emotional Intelligence Scale (EIS) by Hyde et al. (2002) is a comprehensive tool designed to measure various dimensions of emotional intelligence, such as self-awareness, empathy, and emotional stability. It consists of 34 items rated on a Likert scale, making it suitable for assessing emotional competencies in diverse populations [5]. The Minnesota Satisfaction Questionnaire (MSQ) by Weiss et al. (1967) is a widely used instrument for evaluating job satisfaction across different dimensions, including intrinsic and extrinsic factors. Both scales are reliable and validated, making them valuable for psychological and organizational research, particularly in understanding workplace behavior and emotional well-being [6]. The researcher ensured a conducive environment for data collection by explaining the purpose of the study, maintaining confidentiality, and encouraging honest responses. Additionally, informed consent was obtained before administering the questionnaires.

The data was interpreted using a common Likert scale scoring method, where each response was assigned a numerical value: completely agree: 5 points, agree: 4 points, undecided: 3 points, disagree: 2 points, and completely disagree: 1 point. Participants’ total score was obtained by summing responses across all statements. The final score was then evaluated against the interpretation scale: 140 or lower indicates very low, 141-156 represents low, 157-167 signifies middle, 168-179 denotes high, and 180 or above reflects very high. This method helps assess individuals’ emotional intelligence and self-awareness based on their responses. The job satisfaction survey employs a Likert scale, assigning numerical values to responses: 1 for very dissatisfied, 2 for dissatisfied, 3 for neutral, 4 for satisfied, and 5 for very satisfied. The total score was obtained by summing responses across 20 statements. The score percentage was calculated as (total score / 100) × 100, with a maximum possible score of 100 and a minimum of 20. Interpretation is based on percentage ranges: below 25% (≤25 points) indicates not satisfied, 26-74% (26-74 points) represents moderate satisfaction, and above 75% (≥75 points) signifies high satisfaction, providing insight into overall job satisfaction.

Data analysis included both descriptive and inferential statistics. Descriptive statistics, such as mean, standard deviation, and percentages, were used to summarize the findings. These methods provided a clear overview of the levels of emotional intelligence and job satisfaction among staff nurses, helping to understand their overall patterns and trends.

## Results

The analysis was divided into three sections: Section I presents the demographic variables, Section II focuses on the analysis of data related to the level of emotional intelligence scores, and Section III examines the analysis of data related to job satisfaction.

Out of 100 participants, the majority of staff nurses, 58 (58%), were aged between 20 and 30. Most were female, 70 (70%), married, 64 (64%), and had 1-10 years of hospital work experience, 68 (68%). A significant proportion of 60 (60%) reported a monthly salary ranging between Rs. 10,000 and 20,000. Regarding educational qualifications, 52 (52%) had completed a general nursing and midwifery (GNM) course. The majority, 79 (79%), were employed as staff nurses in hospitals, with 47 (47%) working in the general ward. The frequency and percentage distribution of samples with the selected demographic variables are shown in Table 1.

**Table 1:** Frequency and percentage distribution of samples with the selected demographical variables. ANM = auxiliary nurse midwife; GNM = general nursing and midwifery; B.Sc.(N) = bachelor of science in nursing; P.B. B.Sc.(N) = post-basic bachelor of science in nursing; M.Sc.(N) = master of science in nursing; ICU = intensive care unit; OT = operation theater; HDU = high dependency unit

Sr. No.	Demographic variables		Frequency (n)	Percentage (%)
1.	Age in years	20-30	58	58
		31-40	23	23
		41-50	19	19
2.	Gender	Male	30	30
		Female	70	70
3.	Marital status	Single	36	36
		Married	64	64
		Widow/widower	0	0
4.	Years of working experience	1-10 years	68	68
		10-20 years	28	28
		More than 20 years	4	4
5	Salary per month (in Rs.)	10,000-20,000	60	60
		20,000-30,000	33	33
		More than 30,000	7	7
6	Professional qualification	ANM	12	12
		GNM	52	52
		B.Sc. (N)	18	18
		P.B. B. Sc. (N)	11	11
		M.Sc. (N)	7	7
7.	Designation	Supervisor	3	3
		Nurse educator	6	6
		In charge	12	12
		Staff nurses	79	79
8.	Working area	ICU	35	35
		General wards	47	47
		Others (OT, HDU)	18	18

The majority of the hospital staff nurses, 77 out of 100, had very high emotional intelligence scores. A total of 13 staff nurses (13%) had high emotional intelligence scores. Seven staff nurses (7%) had moderate emotional intelligence scores working at hospitals. A very low number of staff nurses, 3 (3%), had low emotional intelligence scores working at hospitals. Table 2 shows the frequency and percentage distribution of emotional intelligence scores among staff nurses.

**Table 2:** Frequency and percentage distribution of emotional intelligence levels among staff nurses

Emotional intelligence score	Frequency (n)	Percentage (%)
Very low (140 or lower)	0	0
Low (141-156)	3	3
Middle (157-167)	7	7
High (168-179)	13	13
Very high (180 or above)	77	77

The majority of staff nurses, 63 (63%), working in hospitals reported high levels of job satisfaction, reflecting a positive outlook on their professional environment. Meanwhile, 37 (37%) of staff nurses expressed moderate levels of job satisfaction, indicating room for improvement in certain areas. The distribution of job satisfaction levels among staff nurses is detailed in Table 3.

**Table 3:** Frequency and percentage distribution of job satisfaction among staff nurses

Job satisfaction level	Frequency (n)	Percentage (%)
Not satisfied (<25%)	0	0
Moderately satisfied (26-74%)	37	37
Highly satisfied (above 75%)	63	63

Among staff nurses, the mean emotional intelligence score was 191.59, with a standard deviation of 18.0264. The mean score of job satisfaction was 75.14, and the SD was 11.9974 among staff nurses. At the 5% level of significance, the correlation coefficient between nurses' emotional intelligence and job satisfaction is r = 0.4059, p = 0.000002 < 0.05. The correlation between emotional intelligence and job satisfaction among staff nurses is shown in Table 4.

**Table 4:** Correlation between emotional intelligence and job satisfaction among staff nurses SD: standard deviation

	Mean	SD	r-value	p-value	Correlation
Emotional intelligence	191.59	18.0264	0.4059	0.000002	Positive
Job satisfaction	75.14	11.9974			

## Discussion

This descriptive study aimed to assess the levels of emotional intelligence and job satisfaction among staff nurses working in selected hospitals within the Sangli, Miraj, and Kupwad corporation areas. A total of 100 staff nurses were included in the study to evaluate their emotional intelligence and job satisfaction. The findings revealed a positive correlation between emotional intelligence and job satisfaction, indicating that higher emotional intelligence among staff nurses is associated with greater satisfaction in their jobs. These results highlight the importance of emotional intelligence in enhancing job satisfaction and overall professional well-being among nursing staff.

The study by Judge et al. provides a comprehensive analysis of job satisfaction, highlighting its critical role in workplace dynamics and employee well-being. The findings underscore that job satisfaction is influenced by a combination of individual, organizational, and environmental factors, including personality traits, workplace culture, and leadership styles. Higher levels of job satisfaction are associated with improved performance, reduced turnover, and enhanced mental health among employees. The study emphasizes the importance of fostering positive job attitudes through supportive management practices, opportunities for professional growth, and a healthy work environment to improve overall organizational effectiveness and employee satisfaction [7].

A similar study by Baby and Damodaran examined job satisfaction and organizational commitment among nurses in Kerala. The findings revealed a significant positive relationship between job satisfaction and organizational commitment, indicating that nurses who are more satisfied with their jobs tend to exhibit greater commitment to their organizations. Factors such as supportive work environments, opportunities for professional growth, and recognition of efforts were identified as key contributors to job satisfaction. The study underscores the need for healthcare institutions to implement strategies that enhance job satisfaction, thereby fostering stronger organizational commitment and improving overall workforce stability and performance [8].

The study by Lu, While, and Barriball provides an in-depth analysis of factors influencing job satisfaction among nurses globally. The study identifies key determinants such as workload, professional relationships, remuneration, career development opportunities, and work-life balance. It highlights that job satisfaction is critical to nurse retention, performance, and overall healthcare outcomes. The review emphasizes that addressing organizational and managerial factors, such as fostering supportive leadership and ensuring fair compensation, can significantly enhance job satisfaction. The findings underscore the importance of creating a positive work environment to improve nurse well-being and the quality of patient care [9]. Similarly, the current study also found that workload relationships, salary, and continuing education were found to be significantly influencing job satisfaction.

A similar study by Gui, Barriball, and While explores the effects of job satisfaction among nurse educators and its influencing factors. The study reveals that job satisfaction positively impacts teaching effectiveness, professional commitment, and retention rates among nurse teachers. Key actors affecting satisfaction include workload, organizational support, career progression opportunities, and relationships with colleagues and students. The review highlights the need for educational institutions to address these factors by creating supportive work environments, offering professional development, and balancing teaching and administrative responsibilities. Enhancing job satisfaction among nurse educators is essential for sustaining high-quality nursing education and fostering long-term commitment [10].

The study by Lu, Chang, and Wu examines the interrelationships between professional commitment, job satisfaction, and work stress among public health nurses in Taiwan. The findings reveal that higher professional commitment is associated with greater job satisfaction and reduced work stress. Workload and role conflicts were identified as significant stressors impacting satisfaction levels. The study underscores the importance of fostering professional commitment through supportive management practices and opportunities for career growth. Addressing work stressors and promoting job satisfaction are critical strategies for improving nurse well-being, retention, and the quality of public health services [11]. The findings from the current study suggest that nurses at the administrative level identify similar factors for job satisfaction related to both administrative and patient duties, which aligns with existing literature on the role of emotional intelligence in job satisfaction

In a similar study, Ruggiero explored the relationships between work variables, health factors, and job satisfaction among 247 critical care registered nurses using a descriptive, correlational design. The study revealed that having more weekends off per month, along with lower levels of depression and emotional stress, significantly contributed to higher job satisfaction. The findings emphasize the importance of addressing scheduling practices and mental health concerns to improve nurses' job satisfaction, recruitment, and retention. These insights align with the current research, highlighting the need for targeted interventions to enhance the well-being and satisfaction of nursing professionals [12].

A similar study by Kalliath and Morris explored how job satisfaction impacts burnout among nurses, hypothesizing that higher job satisfaction would predict lower levels of burnout. The findings revealed that job satisfaction had a significant direct negative effect on emotional exhaustion, which in turn positively influenced depersonalization. The study emphasized that improving job satisfaction through organizational efforts, such as addressing stressors and resources, could reduce burnout. This aligns with Ruggiero's (2005) work, which also showed that reducing emotional stress and improving work conditions could enhance job satisfaction and reduce burnout in nurses, further supporting the need for targeted interventions [13]. These studies suggest a strong relationship between emotional intelligence and job satisfaction, as reflected in previous research [10-12].

The study conducted by Hu and Liu examined job satisfaction among nurses in China through a nationwide survey of 403 nurses across 16 provinces. The findings indicated significant dissatisfaction with work, pay, and promotions, with pay being the least satisfying aspect. Nurses with more experience, higher professional titles, and more opportunities for continuing education were more likely to report higher job satisfaction. The study suggests that nurse managers should prioritize improving pay, career advancement opportunities, and recognition of nurses' achievements. Additionally, offering continuing education opportunities and promoting autonomy, critical thinking, and accountability can enhance job satisfaction and retention [14].

A similar study was conducted by Farhana et al. regarding emotional intelligence influences nurses' critical thinking, decision-making, and overall practice. This study aimed to assess the relationship between nurses' job satisfaction and emotional intelligence in tertiary care facilities using a descriptive correlational design. Data was collected through structured self-administered questionnaires and analyzed using SPSS-20. Results showed that 79.2% of participants had high emotional intelligence, and 95.11% were satisfied with their jobs. A significant positive correlation was found (r = 0.240, P = 0.008). The study highlights the importance of emotional intelligence in enhancing job satisfaction and nursing care quality, suggesting its consideration in nursing staff recruitment [15]. Correlation between emotional intelligence and job satisfaction indicates that the emotional state has a positive influence on job satisfaction.

The collective studies emphasize the critical relationship between emotional intelligence, job satisfaction, and professional commitment among nurses. Emotional intelligence is consistently linked to improved job satisfaction, as it enables nurses to manage stress, build positive workplace relationships, and maintain resilience in demanding environments. Organizational factors, including supportive management, opportunities for career development, and balanced workloads, significantly influence satisfaction and commitment. High job satisfaction positively impacts nursing outcomes, retention, and quality of care, while addressing work-related stressors enhances overall performance and well-being.

One limitation of this study is the potential for response bias, as employees may provide socially desirable answers rather than their true feelings, particularly in sensitive areas such as relationships with supervisors or job security. Additionally, the study's reliance on self-reported data may lead to inaccuracies, as individuals may not have a clear or objective view of certain aspects of their job. The use of a fixed 5-point scale may also limit the depth of responses, not capturing the nuances of employees' experiences. Moreover, this study does not account for external factors like personal life circumstances that could influence job satisfaction. Lastly, the sample may not be representative of the entire workforce, limiting the generalizability of the findings to broader populations.

## Conclusions

The study highlights the importance of emotional intelligence as a critical factor influencing job satisfaction among staff nurses. Emotional intelligence equips nurses with the ability to navigate workplace challenges, build strong interpersonal relationships, and maintain a supportive and positive work environment. These skills contribute significantly to enhancing professional satisfaction and improving overall performance. By fostering emotional intelligence through structured training programs and supportive workplace strategies, healthcare organizations can create an environment that promotes greater job satisfaction and better nursing outcomes. Emotional intelligence not only empowers nurses to handle stress effectively but also strengthens their ability to deliver compassionate patient care. Recognizing the pivotal connection between emotional intelligence and job satisfaction is essential for healthcare organizations aiming to ensure a motivated, engaged, and high-performing workforce. Prioritizing the development of emotional intelligence as a fundamental aspect of professional growth can lead to sustained improvements in job satisfaction and the quality of nursing care provided.

## Appendices

A questionnaire designed to assess emotional intelligence and job satisfaction among staff nurses. It includes 50 statements, with response options ranging from "Completely Agree" to "Completely Disagree." The table is structured with columns for serial numbers, statements, and each response category, allowing for clear data collection. The Emotional Intelligence Inventory scale is presented in Table 5.

**Table 5:** Emotional Intelligence Inventory Scale Hyde A, et al. (2002). Emotional Intelligence Scale. National Psychological Corporation [5].

Sr. No	Statements	Completely agree	Agree	Undecided	Disagree	Completely disagree
1.	You can express emotions like happiness, sorrow, anger, etc. at the appropriate time and in appropriate amounts					
2.	You have enough self-confidence					
3.	You find it difficult to withstand criticism					
4.	You are able to produce any expression on your face irrespective of the situation					
5.	You feel lonely					
6.	You have a special skill to solve the conflict between two individuals					
7.	You have the ability to keep secrets					
8.	You are optimistic even in adversity.					
9.	You have the ability to judge correctly other people's views about you					
10.	You ponder over before you act on anything					
11.	You achieve whatever you desire by some means or other.					
12.	You believe that your failure is always due to unfavourable circumstances or undue interventions by others					
13.	You are fully aware of your responsibilities					
14	You become upset when you see other people succeed where you have failed					
15	You have a special skill to adjust to new circumstances					
16	In many situations, other people prefer your leadership					
17	You have the capacity to convert any unfavourable circumstances into favourable ones					
18	Often, you feel that you are unlucky					
19	You consider sympathy and kindness towards others as your major qualities					
20	You can make others accept your viewpoints					
21	It is without your conscious awareness that many a thought enters your mind					
22	You can put across your viewpoints very well to others					
23	You often have feelings of inferiority					
24	You unwillingly disclose everything in your mind to people who are very close to you					
25	You have the habit of making and breaking friendships according to your needs					
26	You break down completely when things happen against your expectations					
27	If in friendship you will love a person as dear as your life, but, if in enmity, you will despise the person to the core					
28	You are able to openly tell anyone whatever you want to say, without feeling shy					
29	You always have a feeling that something untoward is going to happen					
30	You have a knack for understanding other people's mental states					
31	You have the ability to realize your own mistakes and correct them					
32	In crisis situations, you lose your ability to take the right decisions					
33	You take up leadership in almost all the matters in which you are involved					
34	You have a special ability to take along people with different points of view					
35	Whatever the mistakes committed by those who are close to you, you will firmly stand by their side without disowning them					
36	Instead of justifying your own actions, you can take on the perspective of other people for evaluating it					
37	You don’t have any difficulty in accepting your mistakes that are pointed out by others					
38	You feel proud of your friends achievements					
39	You have the ability to maintain friendship stable and steady for long					
40	In order to achieve your goal, you make use of others without their awareness					
41	You have good ability to tide over crisis situations					
42	You are capable of controlling your emotional outbursts.					
43	Other people don’t give you proper recognition					
44	You can express anger without quarrel					
45	You lose your courage at times of crisis					
46	You can very easily settle your differences with others					
47	If you happen to make a mistake, you keep worrying about it for long					
48	Whatever you take up, you always try to do it at your best					
49	Often, other people misunderstand you					
50	You feel that others are exploiting you					

The Minnesota Job Satisfaction Questionnaire includes 20 statements related to different elements of the work experience, such as workload, autonomy, relationship with colleagues, supervisor competence, opportunities for advancement, and job security. Respondents rate each statement on a 5-point Likert scale, ranging from "Very Dissatisfied" to "Very Satisfied." This inventory provides valuable insights into employees' attitudes towards their jobs and work environments, helping organizations identify areas for improvement in employee engagement, motivation, and overall job satisfaction (Table 6).

**Table 6:** Minnesota Job Satisfaction Questionnaire Weiss et al. (1967) [6].

Sr No	At my present job, this is how I feel about...	Very dissatisfied	Dissatisfied	Neutral	Satisfied	Very satisfied
1.	Being able to keep busy all the time.					
2.	The chance to work alone on the job.					
3.	The chance to do different things from time to time.					
4.	The chance to be “somebody" in the community.					
5.	The way my boss handles his\ her workers.					
6.	The competence of my supervisor in making decisions.					
7.	Being able to do things that don't go against my conscience.					
8.	The way my job provides for steady employment.					
9.	The chance to do things for other people.					
10.	The chance to tell people what to do.					
11.	The chance to do something that makes use of my abilities.					
12.	The way company policies are put into practice.					
13.	My pay and the amount of work I do.					
14.	The chance for advancement on this job.					
15.	The freedom to use my own judgement.					
16.	The chance to try my own methods of doing the job.					
17.	The working conditions.					
18.	The way my co-workers get along with each other.					
19.	The praise I get for doing a good job.					
20.	The feeling of accomplishment I get from the job.					

## References

[1] Ibrahim HA, Elgzar WT, Mohamed RE, Salem GM (2016). Relationship between nursing students’ emotional intelligence and their clinical performance during obstetrics and gynaecologic nursing practical training. Am J Nurs Sci.

[2] Mazumder B, Khumyu A, Boonyanurak P (2016). Relationships between organizational commitments, supervisory support and job satisfaction of nurses in a public specialized hospital, Bangladesh. Bangladesh J Med Sci.

[3] Abu Safieh AM, Malak MZ, Abu-Sharour LM, Shehadeh A (2024). Job-related stress, quality of work life, and quality of nursing care among critical care nurses. J Workplace Behav Health.

[4] Munyug TE, Ping K Y, Yuan Fung C (2020). The relationship between emotional intelligence and job satisfaction among state civil servants. International Journal of Service Management and Sustainability.

[5] Hyde A, Pethe S, Dhar U (2001). Publication Manual for Emotional Intelligence Scale. Natl Psychol Corp.

[6] Weiss DJ, Dawis RV, England GW, Lofquist LH (1967). Minnesota satisfaction questionnaire--short form. APA PsycTests.

[7] Judge TA, Zhang SC, Glerum DR (2020). Job satisfaction. Essentials of Job Attitudes and Other Workplace Psychological Constructs.

[8] Baby B, Damodaran DK (2022). Job satisfaction and organizational commitment among nurses in kerala. Mod Trends Multidiscip Res.

[9] Lu H, While AE, Barriball KL (2005). Job satisfaction among nurses: a literature review. Int J Nurs Stud.

[10] Gui L, Barriball KL, While AE (2009). Job satisfaction of nurse teachers: a literature review. Part II: Effects and related factors. Nurse Educ Today.

[11] Lu KY, Chang LC, Wu HL (2007). Relationships between professional commitment, job satisfaction, and work stress in public health nurses in Taiwan. J Prof Nurs.

[12] Ruggiero JS (2005). Health, work variables, and job satisfaction among nurses. J Nurs Adm.

[13] Kalliath T, Morris R (2002). Job satisfaction among nurses- a predictor of burnout levels. J Nurs Adm.

[14] Hu J, Liu H (2004). Job satisfaction among nurses in China. Home Health Care Manag Pract.

[15] Farhana A, Kouser S, Ghani M, Khatoon T, Asghar R (2023). Effect of emotional intelligence on job satisfaction among nurses—a descriptive study. Pak J Med Health Sci.

